# An Antiphospholipid Antibody Profile as a Biomarker for Thrombophilia in Systemic Lupus Erythematosus

**DOI:** 10.3390/biom13040617

**Published:** 2023-03-30

**Authors:** Ryo Hisada, Tatsuya Atsumi

**Affiliations:** Department of Rheumatology, Endocrinology and Nephrology, Faculty of Medicine and Graduate School of Medicine, Hokkaido University, N15W7, Kita-Ku, Sapporo 060-8638, Japan

**Keywords:** antiphospholipid antibody, systemic lupus erythematosus, thrombophilia

## Abstract

Despite recent advances in treatment and significant improvements in prognosis, thrombosis remains the major cause of death in systemic lupus erythematosus (SLE). Antiphospholipid antibodies (aPL) are the main triggers of thrombosis in patients with SLE, with a frequency of approximately 30–40%. Lupus anticoagulant, anticardiolipin, and anti-β2-glycoprotein I antibodies, which are included in the criteria for antiphospholipid syndrome, and ‘non-criteria’ aPL such as anti-phosphatidylserine/prothrombin complex antibodies, are risk factors for thrombosis in patients with SLE. Multiple positivity for aPL is also associated with an increased risk of thrombosis, and scores calculated from aPL profiles can predict the risk of developing thrombosis. Although there is insufficient evidence for treatment, aPL-positive SLE patients should/may be treated with anticoagulants and/or low-dose aspirin as appropriate. This review summarises the evidence on the clinical significance of the aPL profile as a biomarker of thrombophilia in patients with SLE.

## 1. Introduction

Thrombosis is a significant and life-threatening complication in systemic lupus erythematosus (SLE). Generally, the prevalence of vascular events in patients with SLE ranges between 10 and 30%, with symptomatic coronary artery disease (CAD) occurring in 6–20% of cases, stroke in 2–15%, and subclinical CAD in 30–40% of patients [1]. Arterial thromboembolism is estimated to occur in 5.1–8.5% of patients within five years of SLE diagnosis [2], and cerebral and cardiovascular ischaemic events are particularly major causes of irreversible disability and death in SLE patients. Venous thrombosis is also known to be increased in SLE patients, occurring in 3.7–10.3% within five years of SLE diagnosis [2]. In a study of 4863 Canadian SLE patients, the multivariate hazard ratios for pulmonary artery thrombosis and deep vein thrombosis were higher than in non-SLE patients; 3.04 (95% CI: 2.08–4.45) and 4.46 (95% CI: 3.11–6.41), respectively [3].

Antiphospholipid antibodies (aPL) are autoantibodies that target a variety of phospholipid-binding proteins and are risk factors for thrombosis and recurrent fetal death [4,5]. An analysis of thrombotic risk factors in SLE patients revealed that age, duration of illness, smoking, aPL positivity, nephritis, and the use of immunomodulatory drugs were identified as risks, with aPL positivity being an extremely high-risk factor, with an odds ratio (OR) of 3.22 [6].

Antiphospholipid syndrome (APS) is defined by venous, arterial, and small vessel thrombosis and obstetric morbidity associated with persistent aPL detected on two or more occasions at least 12 weeks apart [7]. Previous basic research has shown that aPL induces tissue factor expression and procoagulant activity in monocytes and endothelial cells [8,9]. Other mechanisms include aPL-inducing platelet activation [10], complement activation via alternative and classical pathways [11], antagonistic effects on specific components of the coagulation system, such as activated protein C and antithrombin [12,13], and natural killer cell activation [14]. In addition, aPL have recently attracted attention due to growing evidence that they exacerbate the effects on thrombosis via increased neutrophil extracellular traps (NET) formation and impaired NET degradation [15]. Although aPL are pathogenic autoantibodies, they do not necessarily cause thrombosis. Therefore, a ‘two-hit theory’ has been proposed, i.e., that a second trigger, such as an infectious or inflammatory disease, is required for clinical manifestations to develop thrombosis [16].

Graham Hughes first reported APS in 1983 in a group of SLE patients with clinical features of LA and recurrent thrombosis [17]. The prevalence of antiphospholipid antibodies (aPL) in healthy individuals is estimated to be between 1 and 5%, whereas in SLE patients it is significantly higher, around 30–40% [1]. Although not all patients develop thrombosis, thrombotic events can occur in 50–70% of patients with SLE and aPL during 20 years of follow-up [18]. Ruiz-Irastorza et al. reported that cumulative survival at 15 years was lower in SLE patients with APS than in those without APS (65% vs. 90%, *p* = 0.03) [19]. An 8-year prospective observational study of 54 SLE patients also found that, although aPL levels fluctuated over time, patients tended to remain positive or negative for aPL [20], indicating that the risk of thrombosis is not transient. Therefore, the accurate assessment of the aPL profile as a biomarker for thrombophilia and the appropriate prophylactic treatment are important issues in patients with SLE.

## 2. Antiphospholipid Antibodies as a Biomarker for Thrombophilia

The major antigen targets of aPL are β2GPI and prothrombin, yet antibodies directed against many other antigen specificities have been reported. According to the international consensus [7], three aPL tests (criteria aPL) are included in the APS classification criteria. These include both quantitative immunoassays for the detection of IgG and IgM isotypes of anticardiolipin antibodies (aCL) and anti-beta2-GPI antibodies (aβ2GPI), and a phospholipid-based blood coagulation assay for the dysfunction caused by these antibodies, known as the lupus anticoagulant (LA) assay (Table 1).

On the other hand, among patients who are negative for all criteria aPL but present with APS symptoms, the role of ‘non-criteria’ aPL has become apparent in recent years. The autoantigen specificities of these aPL comprise various phospholipids, phospholipid-binding proteins, and coagulation factors [21]. Representative non-criteria aPL include anti-phosphatidylserine/prothrombin complex (aPS/PT), domain I specific aβ2GPI, IgA isotype aβ2GPI, anti-annexin V, and anti-protein S/protein C (Table 1). Some reports suggest that they may be more specific for the diagnosis of APS than the criteria aPL test, and more accurate in predicting the occurrence of thrombotic events. However, they are not yet included in the APS test criteria due to a lack of standardisation of measurement methods and insufficient clinical trials. In the future, these aPL may play a pivotal role in predicting the risk of thrombosis. The risk of thrombosis for each criteria and non-criteria aPL in SLE patients is described below.

## 3. Antiphospholipid Antibodies in SLE Patients

### 3.1. Criteria aPL

#### 3.1.1. Lupus Anticoagulant

LA is a functional test that detects immunoglobulins that induce the phospholipid-dependent prolongation of coagulation time in vitro. The evaluation of LA should be performed using a three-step strategy: (1) a screening test (indicates prolonged coagulation time), (2) a mixed test (excludes coagulation factor deficiency), and (3) a confirmation test (coagulation inhibitor is phospholipid-dependent) [22]. Previous studies have shown that LA positivity is more strongly associated with arterial and venous thrombosis than aCL or aβ2GPI [23]. The Hopkins Lupus Cohort reported that LA was the best predictor of the development of thrombosis in 821 SLE patients [24]. In an analysis of 332 Swedish SLE patients, LA positivity was the only independent risk factor for venous and arterial thrombosis [25]. A meta-analysis by Wahl et al. also found that patients with SLE and LA had an approximately 3.5-fold increased risk of thrombosis (OR 3.56, 95%CI: 2.01–6.30) (arterial thrombosis OR 3.14, 95%CI: 1.41–6.97, venous thrombosis OR 4.89, 95%CI: 2.25–10.64) [26].

LA testing has made significant progress with updated guidelines, but LA testing is still considerably more laborious and complex to perform than immunoassays. Additionally, heparin, vitamin K antagonists (VKA), and direct oral anticoagulants (DOACs) can cause false-positive or false-negative results. In fact, a four-centre Italian study showed that the discordance rate of LA results (discordance in three or more centres) was 45% and was much higher in patients taking VKA, at 75% [27]. Therefore, blood for testing should be taken whenever possible before the administration of these drugs or after a short interruption [28].

#### 3.1.2. Anticardiolipin Antibodies IgG/IgM

Of the series of aPL assays, the immunological detection of aCL was the earliest established. aCL is usually detected by a solid phase assay using a cardiolipin-coated matrix in the presence of bovine serum, with antibodies binding only to CL bound to bovine β2GPI (β2GPI-independent aCL) and those binding to CL bound to bovine β2GPI (β2GPI-dependent aCL). Medium/high titres, required for the diagnosis of APS, are defined as greater than 40 GPL/MPL or the 99th percentile (9). The aCL detected in patients with APS is the β2GPI-dependent aCL, whereas in patients with infections or other autoimmune disorders, a β2GPI-independent aCL may appear and be falsely positive for aCL.

A meta-analysis of APS patients (including those with SLE) revealed that aCL IgG was associated with thrombosis and that the aCL IgG titre correlated with the odds ratio of thrombosis [29]. In another meta-analysis of SLE patients, aCL positivity was associated with a 2.17-fold risk of venous thrombosis (95%CI: 1.51–3.11) and a 3.91-fold risk of recurrent venous thrombosis (95%CI: 1.14–13.38) [26]. Elbagir et al. reported that aCL IgG was significantly associated with venous thrombosis in patients with SLE, but IgM was not [25].

Despite efforts to standardise assays, there is still considerable inter-laboratory variability. New automated chemiluminescent immunoassays (CIA) have been introduced to replace ELISA to improve reproducibility and reduce inter-laboratory variability [30].

#### 3.1.3. Anti-β2-Glycoprotein-I Antibodies IgG/IgM

The existence of autoantibodies against β2GPI was first reported in 1990, when three groups demonstrated that aPL interacts with phospholipids via β2GPI [31,32,33]. β2GPI molecules are composed of five homologous domains in two distinct conformations: closed circular and open conformations. In the circular conformation, where domains 1 and 5 face each other, the binding site for autoantibodies is shielded, whereas in the open conformation there is an epitope in domain 1, where binding of aβ2GPI enhances β2GPI signaling via intracellular ligands [34]. The aβ2GPI are typically detected by solid phase assays using a matrix coated with purified β2GPI, and current classification criteria require antibody titres of 99% or greater [7].

The aβ2GPI play a crucial role in the aetiology of APS and are associated with thrombosis. In particular, aβ2GPI IgG are believed to correlate more strongly with the clinical manifestations of APS than IgM. A large Chinese cohort, including SLE patients, showed that aβ2GPI IgG are the best predictor of arterial thrombosis with an OR of 6.5 (95%CI, 3.64–8.75) [35]. In a study of Swedish SLE patients, aβ2GPI IgG were significantly associated with venous thromboembolism in SLE patients, but IgM were not [25]. A meta-analysis of APS patients in 2003 revealed that aβ2GPI IgG were more consistently associated with thrombosis than IgM [36]. The role of aβ2GPI IgM in SLE patients with aPL is still obscure. A study of 796 SLE patients by Mehrani et al. showed that aβ2GPI IgM did not correlate with arterial or venous thrombosis but were protective against lupus nephritis (OR 0.54, *p* = 0.049) and hypertension (OR 0.58, *p* = 0.008) [37].

### 3.2. Non-Criteria aPL

#### 3.2.1. Anti-Phosphatidylserine/Prothrombin Complex Antibodies IgG/IgM

Prothrombin represents another critical antigenic target for aPL. Antibodies targeting human prothrombin are detected by ELISA by directly coating prothrombin on irradiated plates or by using the phosphatidylserine/prothrombin complex as an antigen, called aPS/PT [38]. The clinical significance of aPT positivity in APS is still debated, while aPS/PT represents a stronger risk factor for thrombosis. Vandevelde et al. studied two groups of patients with autoimmune diseases, including SLE. They found that both aPS/PT IgG (OR 2.72, 95%CI: 1.75–4.24) and aPS/PT IgM (OR 2.81, 1.83–4.32) were significantly correlated with clinical symptoms of APS. Furthermore, aPS/PT was strongly correlated with the presence of LA [39]. A recent meta-analysis of APS patients revealed that 77.4% of LA-positive individuals were aPS/PT positive [40]. Pham et al. also measured aPL in 155 patients with suspected APS (including 15% with SLE) and showed that the specificity of IgG aPSPT for LAC was 100% (96–100%) and IgM aPSPT was 97% (91–100%) [41]. Note that the sensitivity of aPS/PT for LA remained in the range of 40–50%. aPS/PT may be useful as an adjunct diagnosis for LA, as LA requires complex testing techniques and false-positive results are common due to anticoagulant therapy. However, the standardisation of aPS/PT measurement is currently insufficient and awaits further validation.

#### 3.2.2. Domain 1-Specific Anti-β2-Glycoprotein-I Antibodies

Anti-β2GPI antibodies exhibit heterogeneous reactivity towards β2GPI, and the identification of the target domain among the five homologous domains of β2GPI could improve the specificity and utility of the test. De laat showed that antibodies against domain I (an epitope consisting of Gly40 and Arg43) have lupus anticoagulant activity and a strong correlation with thrombosis [42]. In a multicentre study of 511 patients, including SLE, domain 1-specific aβ2GPI IgG (aDI) were associated with a high risk of thrombosis with an OR of 3.5 (95%CI: 2.3–5.4), whereas non-domain I aβ2GPI IgG were not associated with a risk with an OR of 0.4 (95%CI: 0.3–0.6) [43]. A meta-analysis of 1218 APS patients, including 318 SLE patients, showed that aDI were detected in 45.4% of patients and were a significant predictor of thrombosis with an OR of 1.99 (95%CI: 1.52–2.6) [44]. In a recent study of 501 patients with early SLE, aDI IgG were found in 29% and were more common than aCL or aβ2GPI [45]. The combination of aDI IgG and aPS/PT IgG/IgM in patients with suspected APS showed a high positive predictive value for the diagnosis of APS, suggesting the potential of these non-criteria aPL as a first-line test for aPL [46].

#### 3.2.3. Anti-β2-Glycoprotein-I Antibodies IgA

Of the three aβ2GPI isotypes, aβ2GPI IgA are not included among the criteria aPL, yet their utility still remains controversial due to conflicting results from available studies. IgA class antibodies are called mucosal antibodies as they are produced by B lymphocytes in the mucosa. Ruiz-Garcia et al. performed ELISA tests for aβ2GPI IgA in 156 APS patients and found aβ2GPI IgA positivity in 22.4% [47]. Vlagea et al. also studied 314 patients with APS and SLE, and found that only 7.2% of the APS group were positive for aβ2GPI IgA alone, compared to 76.2% of the SLE group. There was no association between aβ2GPI IgA and APS symptoms [48]. In an analysis of 817 SLE patients from the LUMINA and Hopkins cohorts, aβ2GPI IgA-positive patients developed significantly more arterial thrombosis than negative patients (OR 2.2, 95%CI: 1.1–4.4), but there was no significant difference for venous thrombosis (OR 1.6, 95%CI: 0.9–2.8) [49]. A prospective cohort study of 821 SLE patients also found that aβ2GPI IgA were associated with thrombosis (OR 2.00, 95%CI: 1.22–3.3), with a particularly high risk for venous thrombosis (OR 2.8, 95%CI: 1.42–5.51) [24]. On the other hand, Danowski et al. reported that in 418 SLE patients with APS, aβ2GPI IgA were not associated with any symptoms of APS, in contrast to aβ2GPI IgG/IgM [50].

These conflicting results may be due to significant discrepancies in antibodies detection using different solid phase platforms [51]. Further studies are needed to properly assess the role of a2GPI IgA in patients with SLE and APS.

#### 3.2.4. Anti-Annexin V Antibodies

Annexins are a group of Ca^2+^-dependent proteins that bind phospholipids, and annexin V provides a shield against clotting enzymes. It has been theorised that when annexin V interacts with β2GPI-dependent aPL, its anticoagulant effect is impaired, thereby promoting thrombosis [52]. However, the association with thrombosis remains unclear. In a study of 198 patients with APS by De Laat et al., anti-annexin A5 antibodies did not correlate with thrombosis in APS [53]. Conversely, an analysis of 140 SLE patients showed that anti-annexin V antibodies IgG were detected in 19%, and their carriers were significantly associated with arterial and venous thrombosis compared with non-carriers [54].

#### 3.2.5. Anti-Protein S/Protein C Antibodies

Anti-protein S/ protein C antibodies bind to complexes of phospholipids with protein S and protein C and inhibit their activity, thereby reducing the development of thrombosis [52]. The frequency and levels of anti-protein C antibodies were higher in APS patients, and high-avidity anti-protein C antibodies were associated with recurrent venous thrombosis or venous and arterial thrombosis [55]. In an analysis of 156 SLE patients, anti-protein C was detected in 54.5% of patients and correlated with activated protein C resistance. High-avidity anti-protein C antibodies, detected in 26.3% of patients, were associated with developing thrombosis, suggesting that it may be a new risk marker in SLE [56].

### 3.3. Thrombotic Risk Stratification in SLE Patients

Although the presence of a single positive criteria aPL is sufficient to meet the laboratory criteria for the diagnosis of APS, the simultaneous detection of multiple aPL has been demonstrated to identify individuals at elevated thrombotic risk. Specifically, the coexistence of LA, aβ2GPI IgG and/or IgM, and aCL IgG and/or IgM, known as “triple aPL positivity”, is strongly associated with thrombosis, with cumulative thromboembolic event rates of 12%, 26%, and 44% at 1, 5, and 10 years, respectively [57]. This is not limited to the criteria aPL. For example, a study analysing the risk of venous thrombosis by measuring aCL IgG, aβ2GPI IgG, and aDI IgG in 501 SLE patients with early disease found that those with double or triple antibody positivity showed a trend towards increased risk [45]. A longitudinal study of 101 SLE patients over 15 years revealed that the risk of thrombosis gradually increased with the number of positive aPL, reaching a 30-fold higher risk when four antibody tests (anti-PT, aCL, aβ2GPI, and LA) were all positive [58].

Attempts to stratify the thrombotic risk indicated by the aPL profile have been advanced. Otomo et al. [59] proposed the antiphospholipid score (aPL-S), calculated from the positivity and titres of LA, aCL IgG/IgM and aβ2GPI IgG/IgM, and aPS/PT IgG/IgM. The ‘partial aPL-S’ excludes the KCT mixed test (one of the LA tests) and aPS/PT IgG/IgM from the original test. In the validation cohort of patients with autoimmune diseases, including SLE, high aPL-S was an independent risk factor for new thrombotic events, while high partial aPL-S was not associated with thrombotic risk [59]. However, the efficacy of aPL-S and partial aPL-S was subsequently confirmed in two independent cohorts of SLE patients [60,61]. Furthermore, a combination of aPL-S and platelet count could be used to stratify the risk of developing thrombosis in patients with aPL, including SLE patients [62].

On the other hand, Sciascia et al. proposed the Global APS Score (GAPSS), calculated from aPL positivity (aCL, aβ2GPI, aPS-PT, LA) and conventional cardiovascular disease risk factors (hyperlipidaemia, arterial hypertension) [63]. Unlike the aPL-S, which factors in the immunoglobulin type (IgG/M) and titre of each aPL, GAPSS does not take into account these parameters. For example, both high titre aCL IgG and low titre aCL IgM are considered equally aCL positive for thrombotic risk in GAPSS. Similar to aPL-S, GAPSS was significantly higher in thrombophilia cases than in non-thrombophilia cases [63]. Subsequently, the Adjusted Global Anti-Phospholipid Syndrome Score (aGAPSS), which excludes aPS/PT, was developed [64]. In a cross-sectional study using the AntiPhospholipid Syndrome Alliance for Clinical Trials and InternatiOnal Networking (APS ACTION) clinical database and repository, patients with recurrent arterial thrombosis had higher aGAPSS compared to those without recurrence (8.1 ± 2.9 vs. 6.0 ± 3.9, *p* < 0.05) [65]. In 2018, aGAPSS_CVD_ was developed by incorporating the parameters of obesity, smoking habits, and diabetes into the aGAPSS. This resulted in an increased ability to detect cardiovascular disease in patients with aPL compared to the original aGAPSS [66]. The characteristics of these scores are shown in Table 2.

The accurate assessment of thrombotic risk in SLE patients with aPL is crucial for prophylaxis. Nevertheless, both aPL-S and GAPSS are currently of very limited practical application in clinical settings. This is largely attributed to the difficulty of performing all the antibody tests (especially for aPL-S), as well as the lack of standardisation between kits and between centres, making it challenging to disseminate the scores. It is anticipated that future research will lead to more widely accepted scores for classifying thrombotic risk in SLE patients who test positive for aPL.

## 4. The Management of Thromboprophylaxis for SLE Patients with aPL

### 4.1. Primary Thromboprophylaxis

Patients with SLE who have aPL are at increased risk of thrombotic events, and primary thromboprophylaxis should be considered. First, any other hereditary or acquired risk factors for thrombosis should be identified and minimised. In addition to standard cardiovascular risk modification, the management of SLE disease activity is also important [67]. Several prospective cohort studies of aPL-positive SLE patients without a history of clinical thrombosis showed that primary prevention reduced the incidence of thrombosis [68,69]. In a meta-analysis of five randomised clinical trials by Arnaud et al., low-dose aspirin (LDA) use was associated with a significantly reduced risk of first and arterial thrombosis [70]. A Cochrane review by Bala et al. found insufficient evidence of benefit or harm in primary prevention, but the combination of anticoagulants and aspirin was associated with an increased incidence of minor bleeding (epistaxis, dysmenorrhoea) compared with aspirin alone [71].

The European League Against Rheumatism (EULAR) [72] recommends prophylactic treatment with LDA in asymptomatic SLE patients with a ‘high-risk’ aPL profile (defined as the presence of persistent LA, double or triple aPL positivity, and persistent high aPL titre) with or without traditional risk factors. Furthermore, LDA may be considered in SLE patients with a ‘low-risk’ aPL profile (defined as isolated aCL or aβ2GPI at low-medium titres, particularly if transient). Given that prophylactic LDA may be associated with an increased risk of intracranial haemorrhage in healthy subjects [73], the question of whether all SLE patients with a low-risk aPL profile and no history of clinical thrombosis should receive a LDA is controversial. The balance of potential risks and benefits should be explained and discussed with the patient in order to jointly decide on a treatment plan, for which scores such as aPL-S or GAPSS may prove useful.

Hydroxychloroquine (HCQ) is the first-line treatment for SLE and has been demonstrated to have a beneficial effect on the clinical manifestations and long-term prognosis of SLE. It has been suggested that HCQ may protect against thrombosis, for example by inhibiting platelet aggregation [74]. A prospective cohort study of aPL-positive SLE patients without a history of clinical thrombosis revealed a positive correlation between the duration of HCQ use and protection against thrombosis [69].

A cross-sectional study of APS patients uncovered a reduced incidence of thrombosis among those treated with aspirin or HCQ, regardless of concurrent SLE diagnosis [75], suggesting their efficacy in primary and secondary thromboprophylaxis. A randomised controlled trial, designed to prospectively evaluate the potential of HCQ in aPL carriers without systemic autoimmune disease, was prematurely terminated due to logistic reasons and low participation [76]. Another randomised prospective pilot study showed that the combination of HCQ and standard therapy reduced the incidence of thrombosis in both APS patients and aPL carriers over a follow-up period of 2.6 years. This study also revealed that long-term HCQ was associated with a reduction in all aPL titres except IgM aCL [77]. Although the evidence is still insufficient, it has been suggested that the use of HCQs in SLE patients with aPL may be beneficial in terms of their antithrombotic effects.

Statins are widely used in the treatment of hypercholesterolaemia and in the secondary prevention of atherosclerotic disease. Although some basic research suggests that aβ2GPI-mediated endothelial activation is inhibited by statins [78], there is a lack of clinical evidence to support the use of statins in aPL-positive patients with or without SLE. According to the APS treatment guidelines [72], statins are not recommended for primary thromboprophylaxis for aPL/APS patients without hyperlipidaemia, in accordance with guidelines for the general population. Further clinical trials are needed to investigate the effect of statins on thrombosis prevention in SLE-APS patients without hypercholesterolaemia.

### 4.2. Secondary Thromboprophylaxis

Thromboprophylaxis in patients who have developed thrombosis and are diagnosed with APS is similar, regardless of the presence of SLE. For patients with arterial thrombosis, treatment with VKA with INR 2–3 or INR 3–4 is recommended, depending on the individual’s risk of bleeding and recurrent thrombosis. Treatment with VKA with INR 2–3 plus LDA may also be considered [72]. In patients with recurrent arterial thrombosis despite adequate treatment with VKA, the INR target should be raised to 3–4, or the addition of LDA or switching to low molecular weight heparin (LMWH) should be considered, after assessing other potential causes. Combination therapy with HCQ or statins may also be considered [72]. For patients with venous thrombosis, anticoagulation with an INR of 2.0–3.0 is recommended. Patients with recurrent venous thrombosis during VKA treatment should be considered for investigation and education about the adherence to VKA treatment, in addition to frequent INR testing. If an INR target of 2–3 is achieved, the addition of LDA, the increase of the INR target to 3–4 or a change to LMWH may be considered [72].

For patients with venous thromboembolism without aPL, DOACs are the first-line agents, offering improved convenience, non-inferior efficacy, and low bleeding risk [79]. DOACs are attractive for patients with thrombotic APS because of the need for long-term, often lifelong, anticoagulation. However, two randomised controlled trials showed a higher incidence of arterial thrombosis with DOACs compared with VKA, particularly in triple-aPL positive patients [80,81,82]. Based on these trials, international guidelines recommend that DOACs should not be the anticoagulant of choice in high-risk patients (triple-positive, arterial thrombosis, small-vessel thrombosis or organ involvement, or valvular heart disease, according to Sydney criteria) [83]. In low-risk patients with thrombotic APS, DOACs may be an option for anticoagulants, but data to guide management are limited. Their risks, benefits, and uncertainties should be discussed with the patient, and a shared decision-making approach should be taken. Furthermore, there is currently little evidence to support the active use of DOACs in SLE/APS patients, who are at higher risk of thrombosis than non-SLE patients. Regarding the duration of anticoagulant therapy, the current general consensus is that thromboprophylaxis should be continued indefinitely after the onset of thrombosis, given the persistent risk of thrombosis in patients with SLE/APS [18].

Immunosuppressive agents, including biologics, are widely used in patients with autoimmune diseases, yet evidence in the treatment of APS is limited, with only case reports and case series describing positive responses to rituximab and belimumab in patients with refractory APS symptoms [84]. The current guidelines do not generally recommend the use of immunosuppressive agents, except in exceptional cases such as catastrophic APS. However, there is a high unmet need for the treatment of refractory cases that do not respond to anticoagulant therapy, which is an important issue for future research.

## 5. Conclusions

The presence of aPL is a notable risk factor for thrombosis. Accumulating evidence suggests that not only criteria aPL but also some non-criteria aPL are strongly associated with thrombophilia in SLE patients. Furthermore, scoring derived from aPL profiles, such as aPL-S and aGAPSS, is emerging as a means to stratify thrombosis risk. The standardisation of aPL testing, the clarification of the role of non-criteria aPL, the improved utility of aPL profile scoring for thrombotic risk assessment, and new clinical trials on thromboprophylaxis will improve the prognosis of SLE patients with aPL. Further research in the future is eagerly awaited.

## Figures and Tables

**Table 1:** The list of criteria and representative non-criteria aPL.

Criteria aPL	LA aCL IgG/IgM aβ2GPI IgG/IgM
Representative non-criteria aPL	aPS/PT IgG/IgM Domain 1-specific aβ2GPI IgG aβ2GPI IgA Anti-Annexin V antibodies Anti-Protein S/Protein C antibodies

aPL, antiphospholipid antibodies; LA, lupus anticoagulant; aCL, anticardiolipin antibody; aβ2GPI, anti-β2-glycoprotein I; aPS/PT, anti-phosphatidylserine/prothrombin complex.

**Table 2:** The scoring items for aPL profile scores.

Score	aPL Tests	Conventional Cardiovascular Disease Risk Factors	Reference
Antiphospholipid Score (aPL-S) *1	LA (APTT, dRVVT, KCT mixing tests) aCL IgG (high titre *2, medium/low titre) aCL IgM aβ2GPI IgG (high titre, medium/low titre) aβ2GPI IgM aPS/PT IgG (high titre, medium/low titre) aPS/PT IgM	N/A	[59]
			
Partial aPL-S	LA (APTT, dRVVT) aCL IgG (high titre, medium/low titre) aCL IgM aβ2GPI IgG (high titre, medium/low titre) aβ2GPI IgM	N/A	[59]
			
Global Anti-Phospholipid Syndrome Score (GAPSS) *3	LA (score 4) aCL IgG or IgM (5) aβ2GPI IgG or IgM (4) aPS/PT IgG or IgM (3)	Hyperlipidaemia (3) Arterial hypertension (1)	[63]
			
Adjusted GAPSS (aGAPSS)	LA (4) aCL IgG or IgM (5) aβ2GPI IgG or IgM (4)	Hyperlipidaemia (3) Arterial hypertension (1)	[65]
			
aGAPSS_CVD_	LA (4) aCL IgG or IgM (5), aβ2GPI IgG or IgM (4)	Hyperlipidaemia (3) Arterial hypertension (1) Diabetes (2) Obesity (2) Smoking habit (1)	[66]

LA, lupus anticoagulant; APTT, activated partial thromboplastin time; KCT, kaolin clotting time; dRVVT, dilute Russell’s viper venom time; aCL, anticardiolipin antibody; aβ2GPI, anti-β2-glycoprotein I; aPS/PT, anti-phosphatidylserine/prothrombin complex; CVD, cardiovascular disease. *1 The aPL-S is calculated as the total scores for positive aPL tests. The score for each aPL is determined by the odds ratios, as follows: 5 × exp([odds ratios] − 5)/4. *2 High titres are established as more than the median levels of antibody positive patients. *3 GAPSS is calculated as the total score for positive aPL tests and conventional cardiovascular disease risk factors. The risk factors were identified by multivariate analysis weighted points proportional to the β-regression coefficient values (rounded to the nearest integer).

## Data Availability

Not applicable.
